# Antimicrobial Resistance Challenged with Platinum(II) and Palladium(II) Complexes Containing 1,10-Phenanthroline and 5-Amino-1,3,4-Thiadiazole-2(3H)-Thione in *Campylobacter jejuni*

**DOI:** 10.3390/antibiotics11111645

**Published:** 2022-11-17

**Authors:** Meiry Leandra de Lacerda, Daise Aparecida Rossi, Eduarda Cristina Alves Lourenzatto, Micaela Guidotti Takeuchi, Wesley Almeida Souza, Raphael Tristão Cruvinel Silva, Luma Gonçalves Julio, Wendell Guerra, Roberta Torres de Melo

**Affiliations:** 1 Institute of Chemistry, Federal University of Uberlândia, Uberlândia 38400-902, MG, Brazil; 2 Laboratory of Molecular Epidemiology, Federal University of Uberlândia, Uberlândia 38402-018, MG, Brazil; 3 Institute of Exact and Earth Sciences, Federal University of Mato Grosso, Pontal do Araguaia 78698-000, MT, Brazil

**Keywords:** bacterial resistance, *Campylobacter jejuni*, metal-based drugs, platinum(II) complexes, synergism

## Abstract

**Highlights:**

**What are the main findings?**

- Two new complexes containing mixed ligands were prepared and characterized;
- NMR data indicated that two thiolate ligands coordinate a metal ion via the sulphur atom after deprotonation.

**What is the implication of the main finding?**

- The platinum complex was active against three strains of *Campylobacter jejuni*;
- A synergistic effect was observed when the Pt (II) complex was combined with ciprofloxacin.

**Abstract:**

This work describes the synthesis and characterization of two metal complexes of the type [M(L_1_)_2_(phen)], where M = Pt^2+^ (complex **I**) or Pd^2+^ (complex **II**), L_1_ = 5-amino-1,3,4-thiadiazole-2(3H)-thiolate and phen = 1,10-phenanthroline. The in vitro antibacterial activity of these complexes was investigated in isolation and synergistically with ciprofloxacin (CIP) and erythromycin (ERY) in three strains of *Campylobacter jejuni* (MIC = 32 mg/L for CIP and ERY), selected from a bank of 235 strains representative of three poultry exporting states of the country (A, B and C), previously analyzed for epidemiology and resistance to CIP and ERY. A total of 53/235 (22.55%) strains showed co-resistance to CIP and ERY. Isolated resistance to CIP was higher than to ERY. Epidemiological analysis showed that resistance to CIP was more evident in state B (*p* < 0.0001), as well as a higher susceptibility to ERY in state C (*p* = 0.0028). Co-resistance was expressive in state A and in the spring and fall seasons. The evaluation of **I** alone and in synergy with CIP and ERY found values up to 0.25 mg/L not significant for ERY. Complex **II** did not show an antimicrobial effect on the three strains of tested *C. jejuni*. The effect provided by complex **I** represents a promising alternative for control of resistant strains of *C. jejuni*.

## 1. Introduction

Infections caused by antimicrobial-resistant bacteria represent one of the greatest threats to global health, accounting for approximately 1.2 million deaths per year from a worldwide perspective [1,2]. *Campylobacter jejuni* is the most prevalent zoonotic bacterial pathogen associated with foodborne gastroenteritis worldwide and represents a major recent challenge due to the increasing development of outbreaks associated with strains resistant to the drugs of choice for treatment, which include fluoroquinolones and macrolides. Although *Campylobacter jejuni* and Campylobacter coli are the species most often implicated in carcasses, other thermotolerant species, such as Campylobacter lari and Campylobacter upsaliensis cause campylobacteriosis and are foodborne [3,4,5].

In the search for new antimicrobial agents, the most promising strategy aims to develop novel agents with new mechanisms of action and new cellular targets [6]. In this sense, metal complexes have access to unique modes of action and such mechanisms are difficult if not impossible to replicate with purely organic compounds [7]. In addition, the use of metal complexes demonstrates therapeutic value and well-documented pharmacological applications [8] for genera such as Salmonella, among others [9,10,11,12,13,14].

Indeed, the configuration of metal complexes allows the adoption of distinct geometries and oxidation states, which reflect in different modes of action on the pathogen [15]. This degree of flexibility makes them attractive in antimicrobial therapy [16] and their association with phenanthroline derivatives have been shown to possess promising activity in disrupting the metabolism of metals crucial for microorganisms, such as interfering with the acquisition and bioavailability of essential ions. Moreover, the low toxicity profile of these compounds consolidates their potential for clinical approval, including studies with antineoplastic activity [17]. For example, several metal complexes have been screened by the Community for Open Antimicrobial Drug Discovery (CO-ADD) for antimicrobial activity. When compared to organic drugs, the metal complexes showed a significantly higher hit-rate not exhibiting any cytotoxicity against mammalian cell lines or hemolytic properties, especially the metal complexes of ruthenium, silver, palladium, iridium and platinum [7], which justifies the design and development of metal complexes as antimicrobial drugs.

In parallel, many studies have focused on the benefits of drug combinations to act at different target sites in bacterial cells to enhance the efficacy of traditional antimicrobials. Synergistic therapy has advantages that include reduction in treatment time, broadening the spectrum of activity, an increase in drug stability and bioavailability, delay in the emergence of bacterial resistance, reduction in side effects by efficacy at lower concentrations, and restoration of the antibiotic activity of the clinical agent by desensitizing strains to the drugs for which they have become resistant [18].

In summary, examination of synergy can promote suppression of the evolution of antimicrobial resistance and provide the solution in combating difficult-to-control pathogens [19], significantly increasing the effectiveness of traditional antimicrobials associated with metal complexes [20].

By the year 2020, campylobacteriosis accounted for 60% of gastrointestinal zoonoses notifications with more than 120,000 confirmed cases in the European Union [21]. In the United States, it is estimated that approximately 1.5 million people become ill from *Campylobacter* spp. infections every year [22]. In Brazil, there are no confirmed outbreaks of campylobacteriosis due to the absence of notifications and the disarticulation of actions in the health arena, besides the difficulty of isolation and characterization of the genus [23,24].

The importance of Brazil in this context occurs due to its position as the largest exporter of chicken meat, the main vehicle of transmission of the pathogen, with 4,231 tons in 2020 and with a growing productivity, reaching the highest production since 2010. Despite this, there is no official data regarding the contamination of chicken carcasses [24,25,26].

Motivated by the problem regarding the antimicrobial resistance in *C. jejuni*, the country’s prominent position in the export of chicken meat and the promising strategy of using metal complexes, this study aimed to describe the synthesis and physicochemical characterization of two new metal complexes, evaluate the epidemiology of *C. jejuni* resistance representative of the national territory, and determine the isolated and synergistic effect of these new actives in co-resistant strains combined with the analysis of desensitization. The literature has not yet reported studies of antimicrobial activity for 5-amino-1,3,4-thiadiazol-2(3H)-thione or its metal complexes. Then, to the best of our knowledge, this is the first paper to describe the antibacterial properties of platinum(II) and palladium(II) complexes containing 5-amino-1,3,4-thiadiazol-2(3H)-thione.

## 2. Results and Discussion

### 2.1. Chemistry

**Figure 1:**
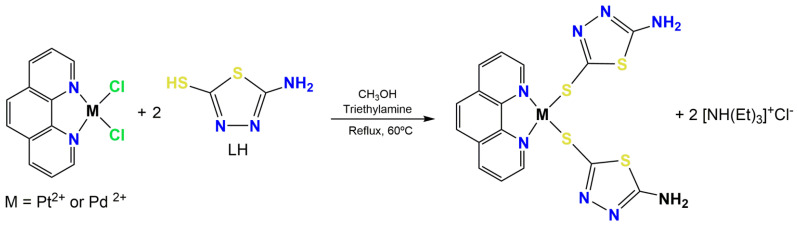
Synthetic route and structure proposed for complex **I** and **II**.

In this work, two new metal complexes were prepared (see Figure 1) under mild conditions with good yields (>80%). In both cases, the metal ion is coordinated to two 5-amino-1,3,4-thiadiazole-2(3H)-thiolate ligands (L_1_) and to a 1,10-phenanthroline molecule. The metal complexes were characterized by elemental analysis, molar conductivity measurements, FT-IR, and nuclear magnetic resonance (^1^H, ^13^C, and ^195^Pt NMR). Both complexes are stable to air and light and were isolated as orange solids soluble in dimethylsulfoxide (DMSO). The results of the elemental analysis (% CHN) indicate that the compounds have a high degree of purity and the molar conductivity values measured in 1.0 × 10^−3^ M dimethylsulfoxide confirmed the non-electrolytic nature of these complexes. 

As to the spectral data, the infrared spectrum of the 5-amino-1,3,4-thiadiazole-2(3H)-thiol ligand showed two bands at 3327 cm^−1^ and 3246 cm^−1^ attributable to the symmetric stretching ν_s_(NH_2_) and the asymmetric stretching ν_as_(NH_2_) mode of amine group. A band close to 3000 cm^−1^ due to NH stretching was also observed, suggesting the presence of the thione tautomer. In addition, a very weak S-H band at approximately 2760 cm^−1^ reinforces that in the solid state the 5-amino-1,3,4-thiadiazole-2(3H)-thiol ligand is in the thione form [27]. On the other hand, the S-H and N-H bands did not appear in the spectra of the metal complexes. Furthermore, the characteristic bands of thioamides at 1606, 1361, 1174 cm^−1^ observed in the free ligand were not observed in the spectra of the metal complexes, also suggesting deprotonation in the N-H group. These observations corroborate the coordination of the ligand to the metal ions (Pt^2+^ or Pd^2+^) through the sulphur atom in the thiolate form [28].

**Figure 2:**
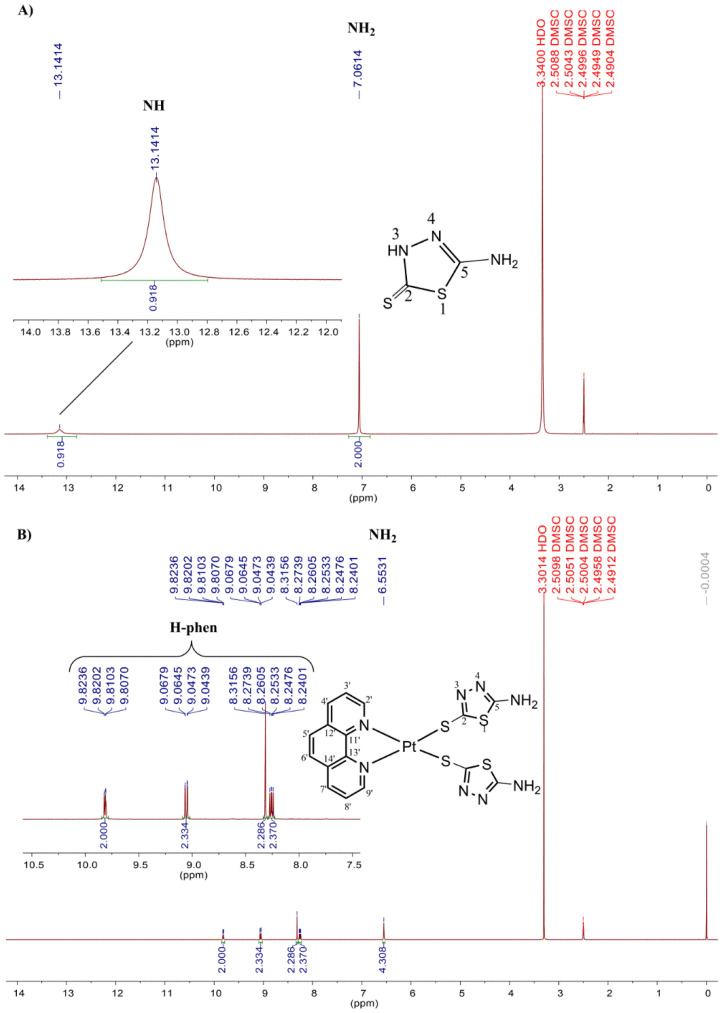
^1^H NMR spectrum (DMSO-*d_6_*, 400 MHz) of the ligand 5-amino-1,3,4-thiadiazole-2(3H)-thione with expansion from 12–14 ppm (**A**) and of the complex **I** expanded from 7.5–10.5 ppm (region assigned to hydrogens of the phenanthroline) (**B**).

**Figure 3:**
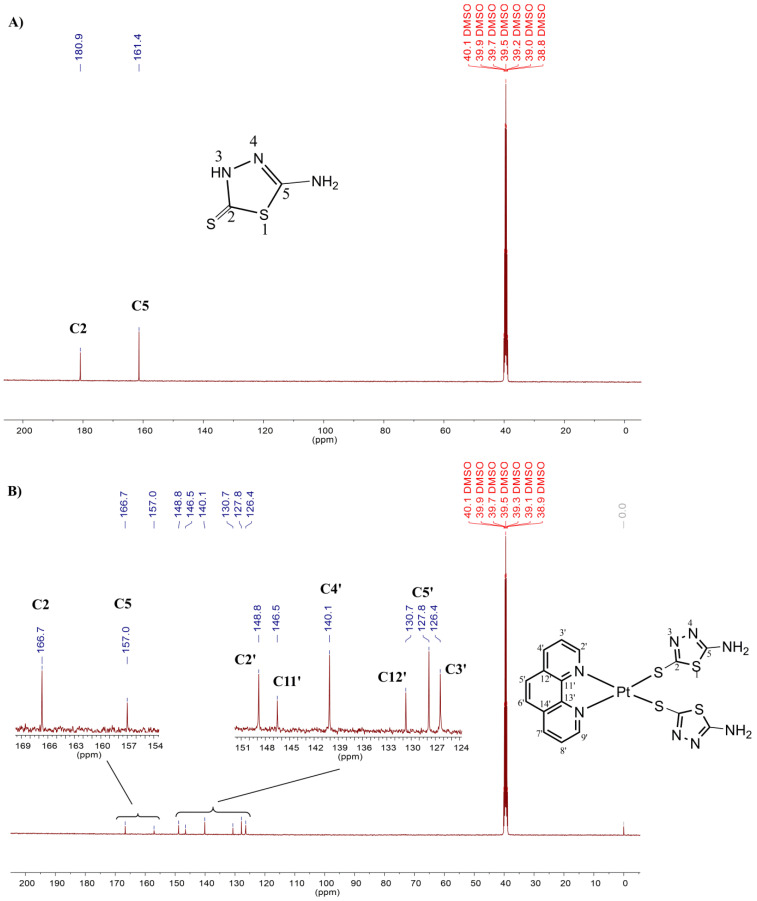
^13^C NMR spectrum (DMSO-*d_6_*, 100 MHz) of the ligand 5-amino-1,3,4-thiadiazole-2(3H)-thione (**A**) and of the complex **I** expanded from 124–151 (region assigned to carbons of the phenanthroline) and between 154–169 ppm (region assigned to carbons of the thiadiazole rings) (**B**).

The ^1^H and ^13^C NMR spectra of complexes **I** (Figure 2 and Figure 3) and **II** (Figures S1 and S2, Supplementary Material) are very similar and only the spectrum of **I** will be discussed. In the ^1^H NMR spectrum of the free ligand, the signals corresponding to the NH_2_ and NH protons appeared as a singlet at δ7.06 and 13.14, respectively (see Figure 2). The NH proton did not appear in the spectrum of complex **I** and the signal corresponding to the NH_2_ protons was very little affected excluding the participation of this group in the coordination. Indeed, an integral value equal to 4 for the NH_2_ group and the absence of the NH proton in the ^1^H NMR spectrum of **I** suggests the presence of two thiolate ligands coordinated to the metal ion via the sulphur atom upon deprotonation (S^−^). In the ^13^C NMR spectrum of complex **I**, it is possible to observe the signals from C2 and C5 carbons at δ 166.7 and 157.0 (Figure 3) while in the spectrum of 5-amino-1,3,4-thiadiazole-2(3H)-thiol these signals were observed at δ 180.9 and at 161.4 ppm, respectively. Thus, in the ^13^C NMR spectrum of complex **I**, the most strongly downfield-shifted signal was the C2 carbon, which also indicates that one platinum ion is coordinated to the ligand via the sulphur atom upon deprotonation [28]. As to the ^195^Pt NMR spectrum of complex **I**, a signal at –3557 ppm is in agreement with the PtN_2_S_2_ coordination sphere (see Figure S3, Supplementary Material) [29].

### 2.2. Epidemiological and Antimicrobial Resistance Study of the Bank of 235 Strains of C. jejuni

Resistance to CIP (121/235, 51.5%) was significantly higher than ERY (83/235, 35.3%). Nevertheless, the results of MIC_50_ and MIC_90_ demonstrated the need for expressive concentrations of ERY (4 and 32 mg/L, respectively), being at least two or even four times higher than the MIC_50_ and MIC_90_ identified for CIP (2 and 8 mg/L, respectively) (Table 1).

The frequencies and percentages of the resistance profiles found for the 235 strains of *C. jejuni* are described in Table 2 and broken down according to the epidemiological characteristics evaluated. Of the 235 strains, 53 (22.55%) showed mutual resistance to CIP and ERY, 68 (28.93%) were resistant to CIP only, 30 (12.76%) to ERY only, and 84 (35.74%) were sensitive to both antibiotics. Furthermore, we observed that ERY-only resistance was lower than the other profiles identified (*p* = 0.0075, Fisher’s test) and that the profile including sensitive strains was the most prevalent (*p* = 0.0023, Fisher’s test). 

Resistance to the drugs of choice in the treatment of campylobacteriosis has proven alarming. In the United States, the concern reflects in the healthcare sector with approximately 310,000 cases of potentially untreatable Campylobacter infections, leading to 28 deaths annually [22]. Despite geographic heterogeneity in resistance profiles, the issue is directly associated with antibiotic mismanagement reflected especially in the agricultural sector, and in low-income countries, whose underreporting is significant [30]. In India, resistance to CIP and ERY reaches values of 33.3 and 21.4% in strains isolated from chicken carcasses [31]. However, Poudel et al. (2022), in China, demonstrated a reduction in the levels of resistance to CIP (from 28% to 15.3%) in isolates from chickens raised without the use of antimicrobials in veterinary practices [32]. 

In Brazil, despite finding expressive values, control measures were adopted and resulted in significant mitigation of *C. jejuni* resistance levels to drugs such as ERY (from 38.2 to 9.1%) in a slaughterhouse in the state of Minas Gerais [33,34,35,36]. Quinolone resistance has been associated with the presence of two different mechanisms, which include the presence of cmeABC and point mutations present in the gyrA and gyrB genes [37].

A single mutation at the Tre-86-Ile position in the QRDR of *gyrA* is known to lead to the substitution of the amino acid threonine for isoleucine and is responsible for the elevation of the MIC in strains and considered to be the main mechanism of resistance to fluoroquinolones [38]. Of 121 resistant strains, we have already identified the presence of this mutation in 65 of them (53.7%). For macrolides, erythromycin resistance is mainly caused by mutations in positions A2075G and/or A2074C of domain V of the 23S rRNA gene [39], already identified in 24/83 (28.9%) resistant strains in our study [40].

The presence of the ermB gene encoding 23S rRNA methyltransferase and the cmeABC multidrug efflux pump has also been shown to be involved in acquired resistance to erythromycin [41]. The latter has been identified in 80/235 (34.0%) strains in our study to date, including susceptible strains [40]. The presence of this efflux pump is favorable to the pathogen, not only in the elimination of antimicrobials but also in the excretion of metabolites and bile salts harmful to *C. jejuni* [39,41].

At the epidemiological level, co-resistance was expressive (*p* = 0.0342, Fisher’s test) in state A (29/93, 54.71%) over state C (9/60, 15.00%). Overall CIP resistance (33/68, 48.52%) was higher than ERY (6/30, 20.00%), especially for state B (*p* < 0.0001, Fisher’s test). As well as in state C, where the absence of resistance to ERY (0/60, 0.00%) was a site exclusive factor (*p* = 0.0028). It should be noted that in state A we identified the lowest frequency of strains with profile 4 (susceptible) (19/93, 20.43%).

The local variation of resistance profiles is multifactorial, but studies point out that sites that have better monitoring structuring regarding the use of antimicrobials and more rigorous self-control programs show efficiency in reducing the prevalence of microorganisms and the levels of antimicrobial resistance, including *C. jejuni* [33,42,43]. Regarding seasonality, we observed that the P4 profile was significantly more prevalent in summer (20/41, 48.78%), compared to the P1 (3/41, 7.31%; *p* < 0.0001) and P3 (6/41, 14.63%; *p* = 0.0017) profiles. In the spring, ERY-only resistance (12/132–9.09%) was lower than the other profiles (*p* = 0.0038). Furthermore, spring (*p* = 0.0386) and fall (*p* = 0.0052) were the seasons in which we identified more co-resistant strains (30/132, 22.72% and 18/56, 32.14% respectively).

The seasonality of the microorganism is described in temperate countries [44], with a higher frequency in summer months [45,46]; however, there are studies that highlight the need to evaluate the seasonality in tropical regions, since there are no large temperature variations or well-defined seasons. In Brazil, there are studies that show the influence of seasonality, but there are also cases in which there were no changes throughout the year [47,48]. Studies related to the resistance profile of strains throughout the seasons of the year do not demonstrate the influence of seasonality on antibiotic resistance. Our study observed that the seasons with the highest number of strains were spring (132/235) and fall (56/235).

The resistance profiles identified in the strains from three Brazilian states with relevance in poultry meat production show a worrisome picture, since there is no evidence of resistance to antibiotics. Campylobacter strains present in these profiles are absolutely problematic clinically, especially in immunocompromised patients.

### 2.3. Effect of Metal Complexes

**Figure 4:**
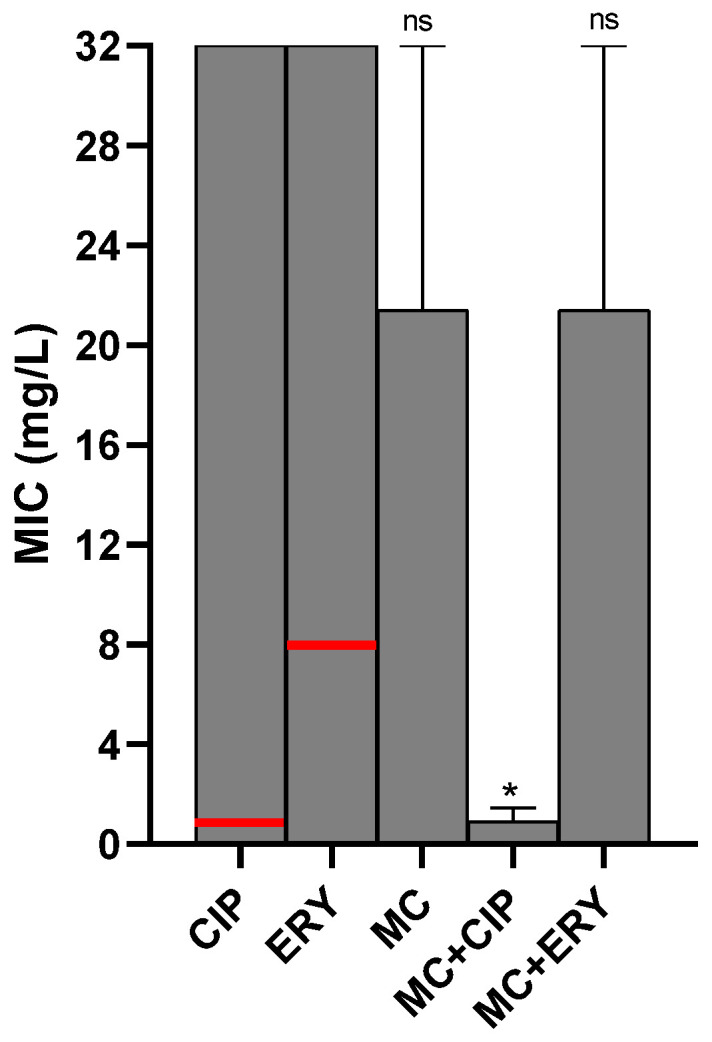
Effect of antimicrobial treatment with and without addition of complex **I** on three *C. jejuni* strains. * *p* < 0.05, ns *p* > 0.05, Mann–Whitney test in comparison with CIP and ERY alone. Red line: cut-off points for ERY and CIP according to CLSI (2021).

The ineffectiveness of macrolides and fluoroquinolones in controlling *C. jejuni* represent a growing public health threat, given its importance as the most prevalent pathogen causing foodborne gastroenteritis worldwide. This demonstrates the importance of studies including promising new substances and combinations on an ongoing basis as treatment options. Figure 4 shows a comparative analysis of the effect of complex **I** on three co-resistant *C. jejuni* strains that had the highest MICs for CIP and ERY (32 mg/L).

Of the three strains tested, we obtained, for the isolated use of complex **I**, MIC values equivalent to 0.25 for one strain and 32 mg/L for the other two. In synergism, complex **I** reduced the MIC of CIP to 0.25, 0.5, and 2 mg/L, and for ERY, the change was only identified in one of the strains, whose MIC was reduced from 32 to 0.25 mg/L. Thus, 2/3 and 1/3 strains were desensitized to CIP and ERY, respectively. The only strain that did not show a change in susceptibility profile in synergism was from state A, with the highest amount of co-resistant strains. The grouped analysis of mean values showed that complex **I** was efficient in lower MIC values both alone (21.4 ± 10.6 mg/L) and in synergy with antimicrobials (+CIP = 0.9 ± 0.5; +ERY = 21.4 ± 10.6 mg/L) compared to CIP and ERY. The difference in mean MIC values was significant in complex **I** in synergy with CIP (*p* = 0.0367).

Analyzing the data, it is possible to affirm that the platinum ion plays a pivotal role in the antibacterial activity reported here, since complex **I** was more effective than complex **II**, which did not affect bacterial growth even at the highest concentration tested (32 mg/L). Thus, our results strongly suggest a potentiating activity of platinum against bacterial cells, considering the concentration used especially related to the synergistic effect. The action of complex **I** on bacterial viability is achieved by the ligand’s destructive cellular mechanisms coupled with the metal’s interference in cellular processes. Its sensitizing effect is mainly directed towards destabilizing the bacterial outer membrane through interaction with electronegative chemical groups that promote increased permeability and disruption of internal processes, allowing greater effect and antibiotic activity [6].

The best synergism came from the coadministration of complex **I** + CIP since it resulted in greater ability to desensitize the strains, with an average 34.8× reduction in the MIC value. For the combination with ERY, despite the desensitization of only one strain, the average reduction in the MIC was 1.5×. These results are compatible with previously reported data, where *Escherichia coli* and *Staphylococcus aureus* resistant to ampicillin and kanamycin were desensitized by employing synergistic antibiotic-metal combinations [49].

The expressive effect on the combination of platinum compounds with CIP has already been described for *M. tuberculosis*, showing lower MIC values than those used for the drugs of choice [50].

For complex **II**, the MIC identified for the three strains was equivalent to the MIC used for the drugs CIP and ERY (32 mg/L), and we did not detect any synergistic effect in its use conjugated to the drugs of choice CIP and ERY. The absence of antimicrobial activity of palladium-based thione complexes was also described by Eğlence-Bakır et al. [51] on gram-negative bacteria. In parallel, it is possible that the diversity of molecular factors already identified in the tested strains, such as the *cmeABC* efflux pump [40], may contribute in the infeasibility of the effect for this complex. Although platinum and palladium are chemically similar, platinum’s primary target is DNA, while palladium complexes bind to proteins leading to DNA damage and consequent cell death. Palladium has greater interaction for ligand exchange and for this reason they also interact easily with other compounds, becoming more toxic due to their greater reactivity [16]. Although, additional studies such as molecular docking or assays of interaction with specific biological targets of the *Campylobacter jejuni* strain are needed to investigate the mechanism of action of complex **I** (platinum centre), as well as justify the inactivity of complex **II** (palladium centre).

No previous studies have been conducted to evaluate the synergistic potential of thione- and phenanthroline-based complexes against *C. jejuni* strains. Reports on the activity of new antimicrobial compounds, alone or in combinations, against *C. jejuni* are limited. 

The results in the three strains tested clearly demonstrate an increase in antibacterial potency, especially in the association of complex **I** with CIP, the breadth of research on which can be further explored. Correlation of these results with future studies on the mechanisms of action involved, in vitro in cell cultures and in vivo, are needed to clarify the applicability of these therapies in clinical practice.

## 3. Materials and Methods

### 3.1. Metal Complexes

The metal precursors [PtCl_2_(phen)] and [PdCl_2_(phen)] used in the synthesis of complexes **I** and **II** were prepared as previously reported in the literature [52,53,54]. All reagents and solvents were purchased from Merck and were used as received. Elemental analyses to determine the percentage carbon, hydrogen, and nitrogen (CHN) were performed on a CHNSO PerkinElmer 2400 Analyzer. Infrared spectra (4000–220 cm^−1^) were performed on a PerkinElmer Frontier MIR spectrometer equipped with an attenuated total reflectance (ATR) sample holder with a diamond crystal. Conductivity measurements were performed using a Tecnopon mCA-150 conductivity meter with UV/HPLC grade dimethyl sulfoxide as solvent. ^1^H, ^13^C and ^195^Pt NMR spectra were performed on a Bruker AscendTM 400 Avance III HD spectrometer (9.2 T) at 400 MHz (^1^H), 100 MHz (^13^C), and 86 MHz (^195^Pt) using DMSO-*d_6_* as solvent at room temperature. Chemical shifts were expressed as δ (in ppm) from the internal reference standard TMS (δTMS = 0.00) and K_2_[PtCl_4_] (^195^Pt NMR).

### 3.2. Preparation of Complexes 

The method used to prepare the metal complexes **I** and **II** is described below. First, 0.250 mmol of 5-amino-1,3,4-thiadiazole-2(3H)-thione was solubilized in 5.0 mL MeOH and added dropwise in 0.125 mmol of [PtCl_2_(phen)] (0.0556 g) or [PdCl_2_(phen)] (0.0447 g), depending on the complex, previously suspended in 5.0 mL of MeOH. After the addition of the 5-amino-1,3,4-thiadiazole-2(3H)-thione ligand, three drops of triethylamine were added to the reaction mixture, which was kept under stirring and reflux (60 °C) for 96 h (platinum complex) or 72 h (palladium complex). Next, orange solids were filtered off, washed with water, methanol and ethyl ether and dried under reduced pressure.

#### 3.2.1. Complex I-[Pt(L_1_)_2_(phen)]

Yield: 84.50%. Color: Orange. Massa Weight (g mol^−1^): 639.66408. Anal. Calc. for [Pt(C_2_H_2_N_3_S_2_)_2_(phen)]: C, 30.04; H, 1.89; N, 17.52%; Found: C, 30.43; H, 2.15; N, 17.37%. ^1^H NMR (400 MHz; DMSO-*d_6_*) δ (ppm): 6.55 (s, 4H, NH_2_); 8.26 (dd, ^3^*J* = 8.0 Hz; ^3^*J* = 5.4 Hz, 2H, H3′ e H8′); 8.32 (s, 2H, H5′ e H6′); 9.06 (dd, ^3^*J* = 8.4 Hz; ^4^*J* = 1.4 Hz, 2H, H4′ e H7′); 9.82 (dd, ^3^*J* = 5.2 Hz; ^4^*J* = 1.4 Hz, 2H, H2′ e H9′). ^13^C RMN (100 MHz; DMSO-*d_6_*) δ (ppm): 126.4; 127.8; 130.7; 140.1; 146.5; 148.8 (phen); 157.0 (C5); 166.7 (C2). ^195^Pt NMR (86 MHz; DMSO-*d_6_*) δ (ppm): −3557. FT-IR spectrum in ATR, ν (cm^−1^): 3241, 3109, 3064, 1603, 1583, 1506, 1429, 1395, 1328, 1311, 1044, 1030, 847, 711, 600, 506, 435, 406, 369, 343, 321, 293, 234. ΛM (10^−3^ M em DMSO) = 1.46 S cm^2^ mol^−1^. 

#### 3.2.2. Complex II-[Pd(L_1_)_2_(phen)]

Yield: 91.27%. Color: Orange. Molar Weight (g mol^−1^): 551.00008. Anal. Calc. for [Pd(C_2_H_2_N_3_S_2_)_2_(phen)]: C, 34.88; H, 2.20; N, 20.34%; Found: C, 34.59; H, 2.31; N 20.17. ^1^H NMR (400 MHz; DMSO-*d_6_*) δ (ppm): 6.67 (s, 4H, NH2); 8.20 (dd, ^3^*J* = 8.2 Hz; ^3^*J* = 5.2 Hz, 2H, H3′ e H8′); 8.30 (s, 2H, H5′ e H6′); 8.97 (dd, ^3^*J* = 8.4 Hz; ^4^*J* = 1.4 Hz, 2H, H4′ e H7′); 9.49 (dd, ^3^*J* = 5.2 Hz; ^4^*J* = 1.4 Hz, 2H, H2′ e H9′) ^13^C NMR (100 MHz, DMSO-*d_6_*) δ (ppm): 126.04; 127.54; 130.27; 140.03; 145.68; 149.60 (phen); 157.71 (C5); 168.11 (C2). FT-IR spectrum in ATR ν (cm^−1^): 3264, 3100, 3053, 1598, 1586, 1422, 1406, 1393, 1317, 1306, 1050, 1025, 842, 713, 604, 502, 426, 407, 393, 338, 314, 254, 242. ΛM (10^−3^ M in DMSO) = 1.04 S cm^2^ mol^−1^. 

### 3.3. Strains

We used 235 strains of *C. jejuni* previously isolated and identified in an exploratory study conducted by the Ministry of Agriculture, Livestock and Supply of Brazil (MAPA) and kept in the culture bank of the Molecular Epidemiology Laboratory of the School of Veterinary Medicine of the Federal University of Uberlândia. The strains were isolated from chicken carcasses from exporting slaughterhouses registered in the SIF (Federal Inspection System) during the period from October 2017 to July 2018, from 43 municipalities belonging to three Brazilian states defined as A, B, and C, which represent 64.1% of the national poultry production [55,56].

### 3.4. Preparations of Strains, Antimicrobial and Metal Complexes

Samples stored in cryoprotectant enriched with UHT milk were reactivated in Campylobacter Agar Base Blood Free (CCDA) (Oxoid^®^) and maintained in microaerophilic (Probac) at 37 °C for 48 h [57]. The typical colonies were then morphologically analyzed for the appearance of curved Gram-negative bacillus on gram stain. 

The antibiotics ciprofloxacin and erythromycin, as well as platinum- and palladium-based compounds (complexes **I** and **II**) were tested. All antimicrobial agents were prepared in a stock solution at a concentration of 64 μg/mL.

### 3.5. Minimum Inhibitory Concentration (MIC) and Bactericidal Concentration (MBC) Test

Antimicrobial susceptibility testing of the strains was performed using the broth microdilution method according to the CLSI description [28]. For the tests with commercial antibiotics (CIP and ERY), Mueller-Hinton broth (MH) was prepared with the addition of Ca^2+^, Mg^2+^, and 5% defibrinated sheep blood (Laborclin^®^), and the same medium with the addition of the stock solution of 64 μg/mL, as well as the bacterial suspension (standardized in sterile 0.85% NaCl). Briefly, the bacterial suspension was standardized at a concentration corresponding to 0.5 on the McFarland scale, and the concentrations of 32, 16, 8, 4, 2, 1, 0.5, and 0.25 μg/mL of the antimicrobials were used. Afterwards, the bacterial suspension was inoculated and the microplates incubated at 37 °C for 48 h. The reading was performed visually with the determination of the MIC as corresponding to the lowest concentration where no turbidity was observed, characterized by the change in coloration of the medium. Additionally, a 10 μL aliquot of each dilution was plated on CCDA to verify bacterial growth (BCC) of the respective dilution well. For all tests, negative controls consisting of the medium without added bacteria were used. The cut-off points (μg/mL) considered to classify the strains as resistant were: CIP > 1 and ERY > 8. MIC_50_ and MIC _90_ were defined as the minimal concentrations of metal compounds that promoted 50 and 90% inhibition, respectively, of the bacterial isolates tested.

### 3.6. Isolated and Synergistic Effect of Metal Compounds

Three strains that expressed resistance profiles to commercial antimicrobials at the highest concentrations (32 μg/mL), genotypically and phenotypically distinct (data not shown) and from different Brazilian states were selected for the tests with the metallic compounds.

The isolated assays with the different metallic compounds were performed as described for the commercial antibiotics in the same concentrations of 32, 16, 8, 4, 2, 1, 0.5, and 0.25 μg/mL. The synergistic effect assays were performed with the commercial antibiotics diluted in the same way mentioned above, with 10 μM of each of the metal compounds evaluated in three different assays at a concentration defined according to the results obtained in a pilot test, corresponding to complex **I** (6.39 μg/mL) and complex **II** (5.51 μg/mL).

### 3.7. Statistical Analyses

The results were tabulated and submitted to descriptive statistics with calculation of the percentage of resistance for each antimicrobial and each resistance profile identified. Comparative analyses were performed using Fisher’s test. All the assays with the metal complexes were done in triplicate and the comparative statistics were done by applying the Mann–Whitney test. The tests were performed using Graph Pad Prism 8.0.1 software, with a 95% confidence interval.

## 4. Conclusions

In this work, two new metal complexes were prepared and characterized by conventional techniques. Their structures present two thiolate ligands coordinated in a monodentate manner and a phenanthroline molecule coordinated through its two nitrogen atoms. The metal compounds were tested as antibacterial agents against three strains of *Campylobacter jejuni* and demonstrated the effectiveness of complex **I** action, especially in CIP desensitization.

## Figures and Tables

**Table 1:** Frequency distribution and percentages for the MIC, MIC_50_, and MIC_90_ (mg/L) in C. jejuni isolated from chicken carcasses in Brazil.

Concentration (mg/L)	CIP n (%)	ERY n (%)
0.25	39 (16.59%)	14 (5.95%)
0.5	18 (7.65%)	8 (3.40%)
1	57 (24.25%)	30 (12.76%)
2	60 (25.53%)	42 (17.87%)
4	33 (14.04%)	40 (17.02%)
8	20 (8.51%)	18 (7.65%)
16	4 (1.70%)	5 (2.12%)
32	4 (1.70%)	78 (33.18%)
**R (%)**	121 ^a^ (51.48%)	83 ^b^ (35.31%)
**MIC_50_**	2	4
**MIC_90_**	8	32

R (%): number and percentage of resistant strains; n (%): number and percentage of strains distributed in the different concentrations of the antimicrobials tested; highlighted in grey: resistant strains; a,b: *p* = 0.0006, Fisher’s test.

**Table 2:** Antimicrobial susceptibility profile of *C. jejuni* isolated from chicken carcass broken down by state and season.

Epidemiological Factor	Antimicrobial Resistance Profiles-n (%)				
	Profile 1: CIP/ERY	Profile 2: CIP	Profile 3: ERY	Profile 4: Susceptibility	Total
**Federal State**					
A	29 (31.18%)	21 (22.58%)	24 (25.80%)	19 (20.43%)	93
B	15 (18.29%)	33 (40.24%)	6 (7.31%)	28 (34.14%)	82
C	9 (15%)	14 (23.33%)	0 (0.00%)	37 (61.66%)	60
**Season**					
Spring	30 (22.72%)	43 (32.57%)	12 (9.09%)	47 (35.60%)	132
Summer	3 (7.31%)	12 (29.26%)	6 (14.63%)	20 (48.78%)	41
Autumn	18 (32.14)	12 (21.42%)	9 (16.07%)	17 (30.35%)	56
Winter	2 (33.33%)	1 (16.66%)	3 (50%)	0	6
**Total**	53 (22.55%) ^a^	68 (28.93%) ^a^	30 (12.76%) ^b^	84 (35.74%) ^c^	235

n (%): number and percentage of strains distributed according to the profiles and epidemiological character. Different letters in the same row indicate statistical difference, *p* < 0.05 Fisher’s test.

## Supplementary Materials

The following supporting information can be downloaded at: https://www.mdpi.com/article/10.3390/antibiotics11111645/s1, Figure S1. 1H NMR spectrum (400 MHz, DMSO-*d_6_*) of complex **II**. Figure S2. ^13^C NMR spectrum of **II** (100 MHz, DMSO-*d_6_*) highlighting the region between 125.0 and 170.0 ppm. Figure S3. ^195^Pt NMR spectrum of **I**.
