# Supplementary material for: Antimicrobial Resistance Challenged with Platinum(II) and Palladium(II) Complexes Containing 1,10-Phenanthroline and 5-Amino-1,3,4-Thiadiazole-2(3H)-Thione in Campylobacter jejuni
Source: Antibiotics (Basel). 2022 Nov 17;11(11):1645. doi: 10.3390/antibiotics11111645 (PMC9687049; doi:10.3390/antibiotics11111645)

# Supplementary Material

## Antimicrobial resistance challenged with platinum(II) and palladium(II) complexes containing 1,10-phenanthroline and 5-amino-1,3,4-thiadiazole-2(3H)-thione in *Campylobacter jejuni*

Meiry Leandra de Lacerda[1], Daise Aparecida Rossi[3], Eduarda Cristina Alves Lourenzatto[3], Micaela Guidotti Takeuchi[3], Wesley Almeida Souza[1,2], Raphael Tristão Cruvinel Silva[1], Luma Gonçalves Julio[3], Wendell Guerra[1]* and Roberta Torres de Melo[3]*

[1]Institute of Chemistry, Federal University of Uberlândia, Uberlândia, MG, Brasil

[2]Institute of Exact and Earth Sciences, Federal University of Mato Grosso, Pontal do Araguaia, MT, Brazil

[3]Laboratory of Molecular Epidemiology, Federal University of Uberlândia, Uberlândia, MG, Brasil

*Correspondence: wendell.guerra@ufu.br and roberta-melo@hotmail.com

**Figure S1**. $^1$H NMR spectrum (400 MHz, DMSO-*d6*) of complex **II**.

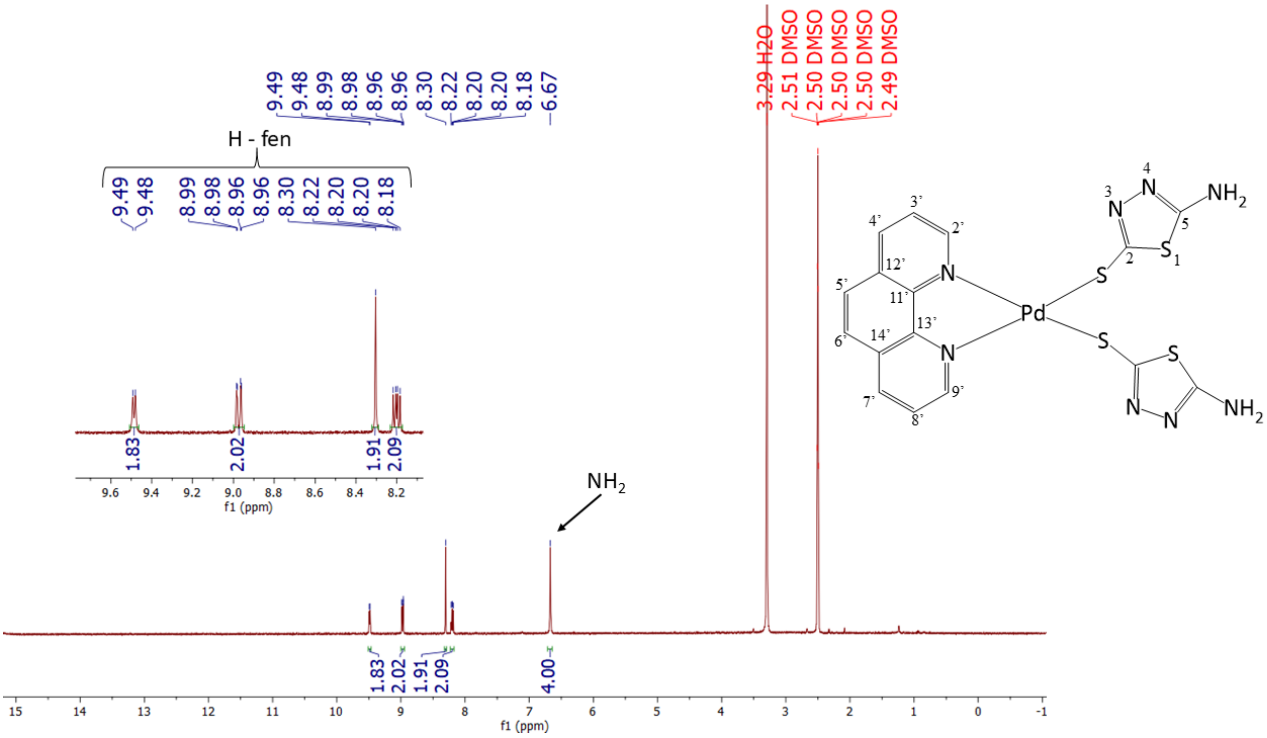

**Figure S2.** $^{13}$C NMR spectrum of **II** (100 MHz, DMSO-*d6*) highlighting the region between 125.0 and 170.0 ppm.

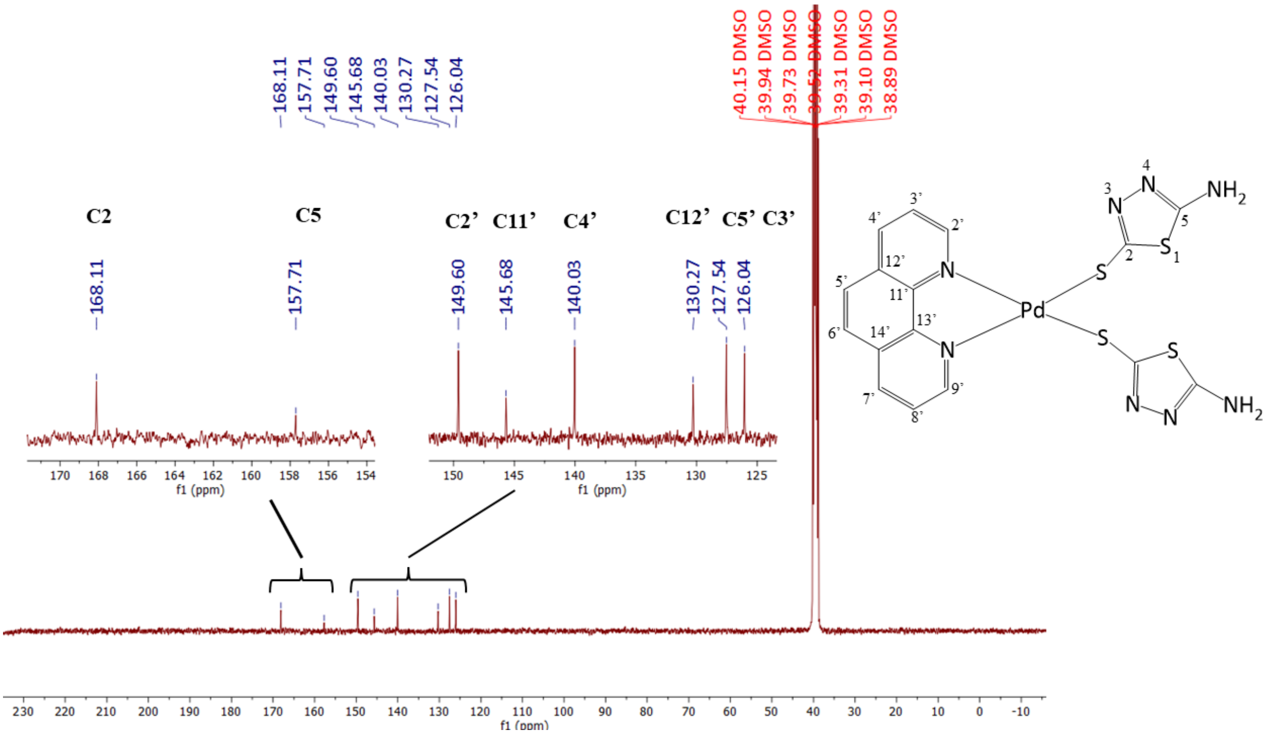

**Figure S3**. $^{195}$Pt NMR spectrum of **I**.

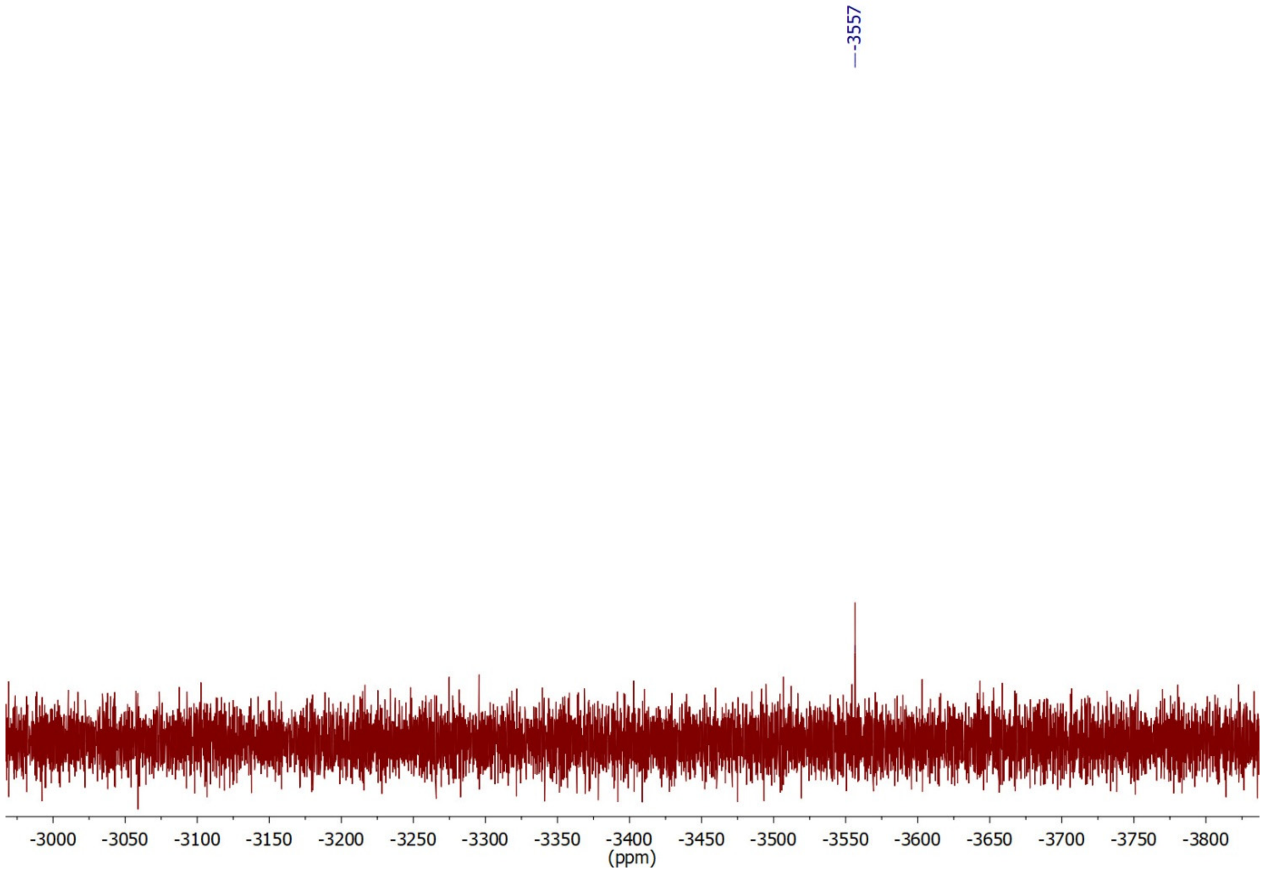

Supplement: Supplementary file 1 [file antibiotics-11-01645-s001.zip › antibiotics-2022824-supplementary.pdf]
